# Systemic capillary leak syndrome complicated by lower extremity compartment syndrome: a case report

**DOI:** 10.1186/s40981-025-00795-8

**Published:** 2025-06-09

**Authors:** Asahi Ishihara, Katsuyuki Sagishima, Tadashi Ejima, Manami Kuwahara, Naoyuki Hirata

**Affiliations:** 1 Department of Critical Care Medicine, Kumamoto University Hospital, Kumamoto, Japan; 2 Department of Anesthesiology, Kumamoto University Hospital, Kumamoto, 860-8556 Japan

**Keywords:** Systemic capillary leak syndrome, Compartment syndrome, Fasciotomy

## Abstract

**Background:**

Systemic capillary leak syndrome (SCLS) is a rare disorder characterized by hypotension, hypoalbuminemia, and hemoconcentration, typically caused by increased vascular permeability due to endothelial dysfunction. We report a case of SCLS complicated by bilateral lower extremity compartment syndrome.

**Case presentation:**

A 29-year-old man developed fever, cough, and rhinorrhea. He was restless, hypotensive, and had generalized edema with tense extremities. Laboratory findings included a hemoglobin level of 24.9 g/dL, hematocrit of 69.3%, albumin of 1.8 g/dL, and creatinine of 3.27 mg/dL. SCLS-induced shock was diagnosed with detection of monoclonal gammopathy of the IgG-λ type. Treatment consisted of fluid resuscitation, vasopressors, high-dose corticosteroids, and intravenous immunoglobulin. Although hemodynamic status improved, he developed bilateral lower-limb compartment syndrome, necessitating fasciotomy. Although the patient exhibited sensory deficits and impaired dorsiflexion and plantarflexion in both ankles, he was able to ambulate with a cane and was discharged on hospital day 50.

**Conclusion:**

This case highlights the risk of serious complications such as compartment syndrome in patients with SCLS.

## Background

Systemic capillary leak syndrome (SCLS) is a rare and potentially life-threatening disorder characterized by three distinct clinical phases: prodromal, fluid extravasation, and recovery [1]. Its etiology remains unknown. The frequency and severity of SCLS episodes vary widely, ranging from a single lifetime event to recurrent attacks occurring multiple times per year [2]. Approximately, 50% of patients experience nonspecific prodromal symptoms 1–2 days before the onset of critical illness, such as oliguria, fatigue, peripheral edema, syncope, abdominal pain, nausea, myalgia, polydipsia, and rapid weight gain [3, 4]. In about 30% of cases, episodes are preceded by upper respiratory tract infections, including COVID-19 [5].

The capillary leak phase typically begins 1 to 4 days following the prodromal period and is characterized by the classical triad of hypotension, hypoalbuminemia, and hemoconcentration, resulting from increased vascular permeability secondary to endothelial dysfunction. Clinically, this phase often resembles septic shock and is managed in a similar fashion [6]. The vascular leak usually resolves within several days, followed by a recovery phase.

Here, we report a case of SCLS complicated by bilateral lower extremity compartment syndrome, an uncommon but serious complication.

## Case presentation

A 29-year-old man (165 cm, 90 kg) with a history of clavicle fracture repair, no regular medications or allergies, and a family history of multiple sclerosis in his sister presented with progressive symptoms. Eight days prior to admission, his younger brother developed influenza. The patient developed fever, rhinorrhea, and cough the following day. While the fever resolved 1 day before admission, he subsequently experienced intense thirst, digital edema, and vomiting. On the day of admission, he became profoundly fatigued and unable to ambulate, prompting emergency transport to a local hospital. On arrival, he was found to be hypotensive, with a systolic blood pressure of 50–70 mmHg and a heart rate of 160 bpm. Laboratory tests revealed acute kidney injury with a creatinine level of 3.03 mg/dL, hypoalbuminemia with an albumin level of 3.1 g/dL, and marked hemoconcentration with a hematocrit of 74% and hemoglobin of 26.6 g/dL. Despite receiving 4 L of intravenous fluids and 2 L of oral intake, his condition remained refractory to initial management. Given the presence of the classic triad—hypotension, hypoalbuminemia, and hemoconcentration—SCLS was suspected, and the patient was urgently transferred to the intensive care unit (ICU) of our hospital.

Upon ICU admission, his neurological status was as follows: Glasgow Coma Scale score E3V5M6 and a Richmond Agitation-Sedation Scale score of +3. The pupils were isocoric, with 3.0 mm in diameter and prompt light reflex. He was severely agitated and non-communicative. Vital signs included a body temperature of 35.2 °C, a heart rate of 170 beats per minute in sinus rhythm, a systolic blood pressure ranging from 50 to 60 mmHg while receiving dopamine at 3 µg/kg/min, a respiratory rate of 32 breaths per minute, and an oxygen saturation of 93% on room air. He exhibited generalized cyanosis, cold extremities, and peripheral edema involving the limbs, while the face and neck remained unaffected. Laboratory data showed further deterioration, with a creatinine level of 3.27 mg/dL, serum albumin of 1.8 g/dL, hematocrit of 69.3%, and hemoglobin concentration of 24.9 g/dL. Arterial blood gas analysis revealed severe metabolic acidosis, with a pH of 7.121, bicarbonate concentration of 3.2 mmol/L, and lactate level of 8.38 mmol/L. Due to worsening agitation and the need for interventions, sedation and endotracheal intubation were initiated. Transthoracic echocardiography demonstrated a left ventricular ejection fraction of 52% with no regional wall motion abnormalities. The diameter of the inferior vena cava measured 11.8 mm during expiration and increased to 17.8 mm during inspiration. After aggressive fluid resuscitation and vasopressor support, systemic blood pressure could be maintained over 90 mmHg. As an additional therapy, high-dose methylprednisolone at 500 mg/day for 3 days was commenced. On hospital day 2, monoclonal gammopathy of the IgG-λ type was detected, prompting initiation of intravenous immunoglobulin (IVIG) at 20 g/day for 5 days, based on reported efficacy in SCLS [7]. In the absence of findings suggestive of plasma cell dyscrasias such as multiple myeloma or immunoglobulin light chain amyloidosis, a working diagnosis of SCLS was established, and the treatment regimen was continued.

**Fig. 1:**
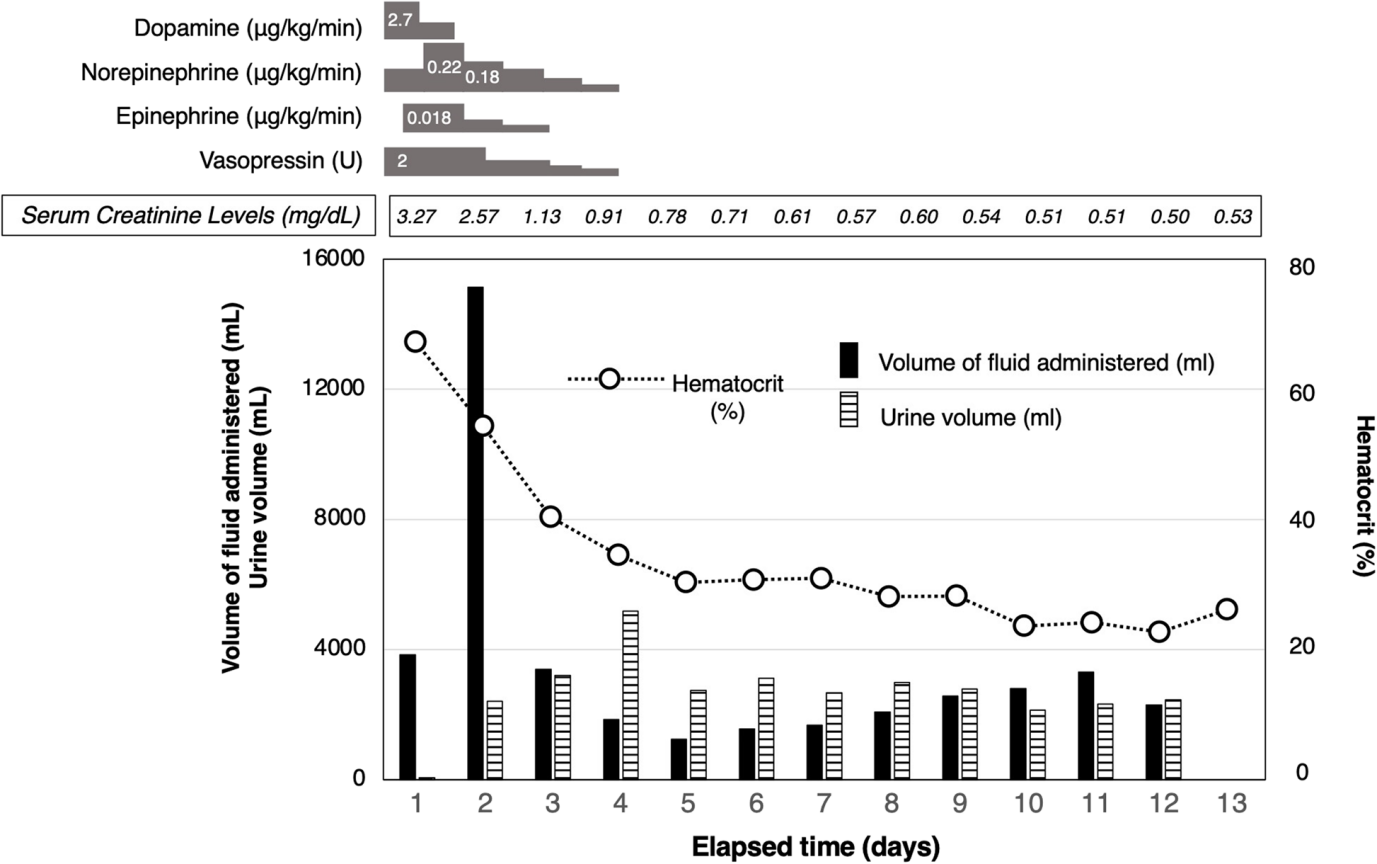
Trends in IV fluids, urine output, hematocrit, catecholamines/vasopressors, and serum creatinine levels

**Fig. 2:**
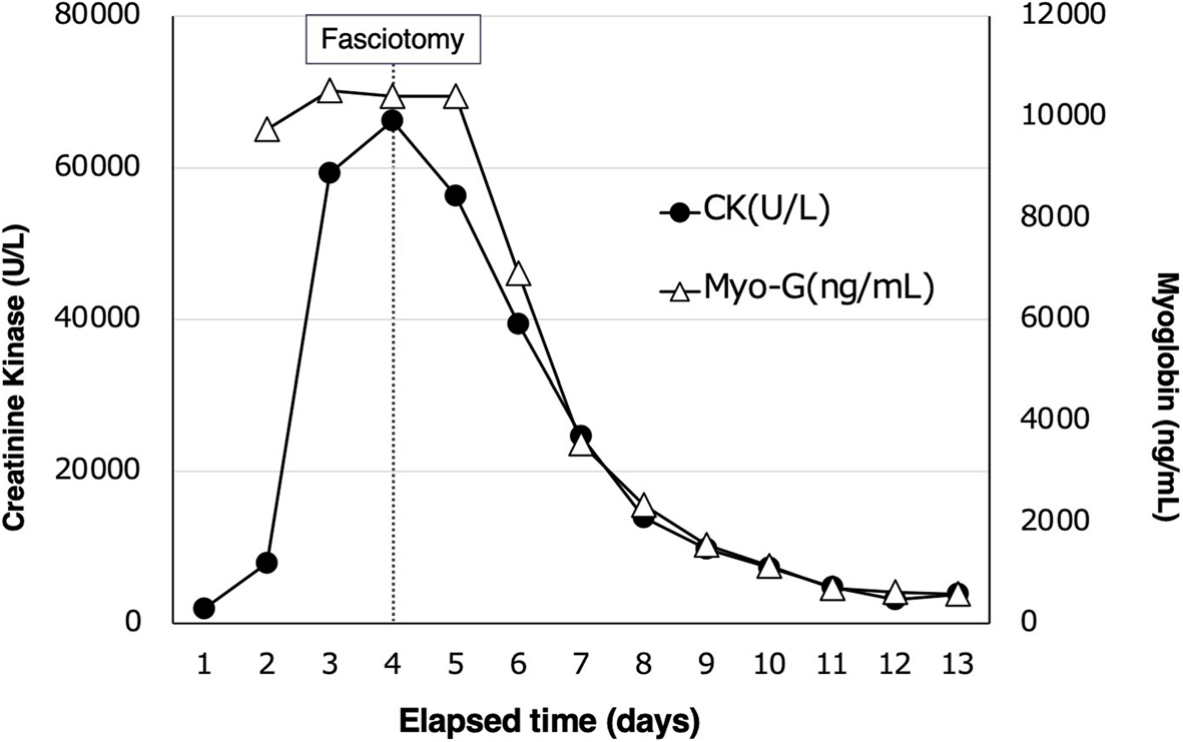
Time course of serum creatine kinase and myoglobin levels. Serial measurements demonstrating the dynamic changes in serum creatine kinase and myoglobin concentrations during the clinical course

**Fig. 3:**
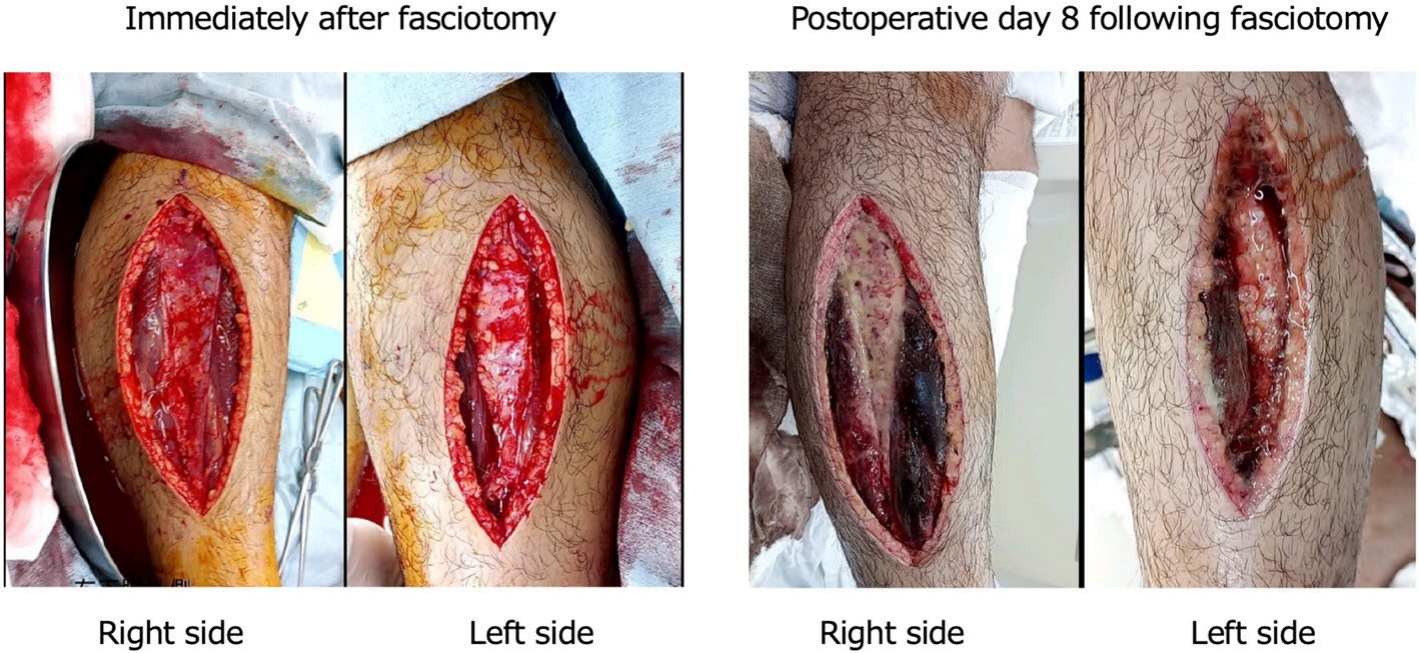
Photographs showing the appearance of the lower legs immediately following fasciotomy on hospital day 4 and on postoperative day 8

From hospital day 3, hemodynamic parameters and hemoconcentration began to improve (Fig. 1). However, progressive swelling of the extremities was observed, accompanied by marked elevations in muscle enzymes: creatine kinase (CK) 59,440 U/L and myoglobin 10,527 ng/mL (Fig. 2). An orthopedic consultation was conducted. In light of the improving urine output and the significant risks associated with fasciotomy—namely infection and neurological complications—conservative management with close monitoring was selected at that time. However, on hospital day 4, CK levels rose to 66,180 U/L, and intracompartmental pressure measurements revealed elevated pressures in the anterolateral compartments of both lower extremities, measuring 46 mmHg on the right and 36 mmHg on the left, so bilateral fasciotomy was performed (Fig. 3).

By hospital day 4, vasopressors and aggressive fluid therapy were discontinued. Diuresis was achieved using intravenous furosemide and continuous infusion of human atrial natriuretic peptide (hANP). With ongoing hemodynamic stabilization and improvement in laboratory parameters, IVIG therapy was completed on hospital day 6, and corticosteroids were tapered. Importantly, no signs of pulmonary edema or heart failure were observed throughout the course.

The patient was extubated on hospital day 12 and transferred to the high care unit the following day. He was subsequently moved to a general ward on hospital day 15. For prophylaxis against recurrence, sustained-release theophylline was initiated at a dose of 200 mg per day, and oral prednisolone was tapered to a maintenance dose of 10 mg per day. Although the patient experienced sensory loss from the bilateral lateral lower leg incision sites to the dorsum of both feet, along with difficulty in dorsiflexion and plantarflexion of both ankles, he was able to walk with a cane and was subsequently discharged on hospital day 50.

## Discussion

Reports of SCLS complicated with compartment syndrome have been limited, and the clinical course varied in each case [8–10]. In previous reports, while SCLS had been diagnosed on the basis of the classic triad of hypotension, hypoalbuminemia, and hemoconcentration, most have either failed to identify monoclonal gammopathy or did so only after the patient’s recovery, and IVIG was initiated in few cases. In contrast, early recognition of monoclonal gammopathy, the IgG in this case, allowed for the early initiation of IVIG therapy on hospital day 2. IVIG has shown promise in both acute management and recurrence prevention [11–13].

Another notable feature of this case is the absence of lower extremity pain both at admission and during the fluid extravasation phase. In previously reported cases of SCLS complicated by compartment syndrome, patients presented with lower limb pain as a chief complaint or developed it early during hospitalization, manifesting during the fluid extravasation phase [8–10]. In contrast, in this case, compartment syndrome became apparent only during the recovery phase. The presentation and progression of compartment syndrome in patients with SCLS can vary considerably between cases.

In our case, the development of compartment syndrome was likely related to aggressive fluid resuscitation during the fluid extravasation phase. Recent studies suggest that a more restrictive fluid management approach may be preferable, as total fluid administration exceeding 10.7 L has been associated with worse outcomes [12]. Nevertheless, it remains unclear whether this finding reflects the detrimental effects of fluid overload or simply correlates with disease severity.

When the compartment syndrome developed in patients with SCLS, the timing of the fasciotomy may warrant reconsideration. In our case, on hospital day 3, the lower limb swelling became apparent; we consulted with the orthopedic team. At that time, since urine output had begun to increase and fasciotomy carries a high risk of infection and neurological complications, a decision was made to closely monitor the patient. However, as the creatine kinase level continued to rise on day 4, fasciotomy was ultimately performed. While the appropriate timing of the fasciotomy remains unclear, multidisciplinary collaboration between intensive care, internal medicine, and surgical teams is essential.

There is currently no standardized treatment for SCLS. In addition to IVIG, agents such as terbutaline and theophylline have been reported to reduce relapse frequency [14, 15]. These drugs are believed to increase intracellular cyclic adenosine monophosphate (cAMP) levels, thereby enhancing endothelial barrier function and reducing capillary permeability [15, 16]. Our patient was started on theophylline for long-term prophylaxis and remained recurrence-free at the time of discharge.

## Conclusion

This case illustrates SCLS complicated by compartment syndrome requiring fasciotomy. Although the diagnosis of SCLS can often be established based on the clinical course and the characteristic triad of hypotension, hypoalbuminemia, and hemoconcentration, early identification of monoclonal gammopathy can facilitate a more definitive diagnosis and enable prompt initiation of intravenous immunoglobulin (IVIG) therapy. Once compartment syndrome develops, close multidisciplinary collaboration among intensive care, internal medicine, and surgical teams is essential for determining an appropriate treatment strategy.

## Data Availability

Data sharing is not applicable to this article as no datasets were generated or analyzed during the current report.

## Abbreviation

COVID-19: Coronavirus disease 2019
